# Supplementary material for: Nanosheets Based Approach to Elevate the Proliferative and Differentiation Efficacy of Human Wharton’s Jelly Mesenchymal Stem Cells
Source: Int J Mol Sci. 2022 May 22;23(10):5816. doi: 10.3390/ijms23105816 (PMC9143505; doi:10.3390/ijms23105816)
Supplement: Supplementary file 1 [file ijms-23-05816-s001.zip › ijms-1735599-supplementary.pdf]

Supplementary information

Nanosheets based approach to elevate the proliferative and differentiation efficacy of human Wharton's jelly Mesenchymal stem cells

Suraj Kumar Singh [1#], Anshuman Singh [1], Vinod Kumar [1], Jalaj Gupta [1], Sima Umrao[2#], Manoj Kumar [3], Devojit Kumar Sarma [3], Marcis Leja[4,5,6], Manohar Prasad Bhandari[4*], Vinod Verma [1]*

[1] Stem Cell Research Centre, Department of Hematology, Sanjay Gandhi Post-Graduate Institute of Medical Sciences, Lucknow - 226014, Uttar Pradesh, India

[2] Indian Institute of Science (IISC), Bangalore - 560012, India

[3] ICMR- National Institute for Research in Environmental Health, Bhopal - 462030, Madhya Pradesh, India

[4] Institute of Clinical and Preventive Medicine, University of Latvia, Riga, LV-1586, Latvia

[5] Faculty of Medicine, University of Latvia, Riga, LV-1586, Latvia

[6] Riga East University Hospital, Riga, LV-1038, Latvia

#Equal Contributor

* Correspondence: vverma29@gmail.com, vverma@sgpgi.ac.in, manoharpbhandari@gmail.com

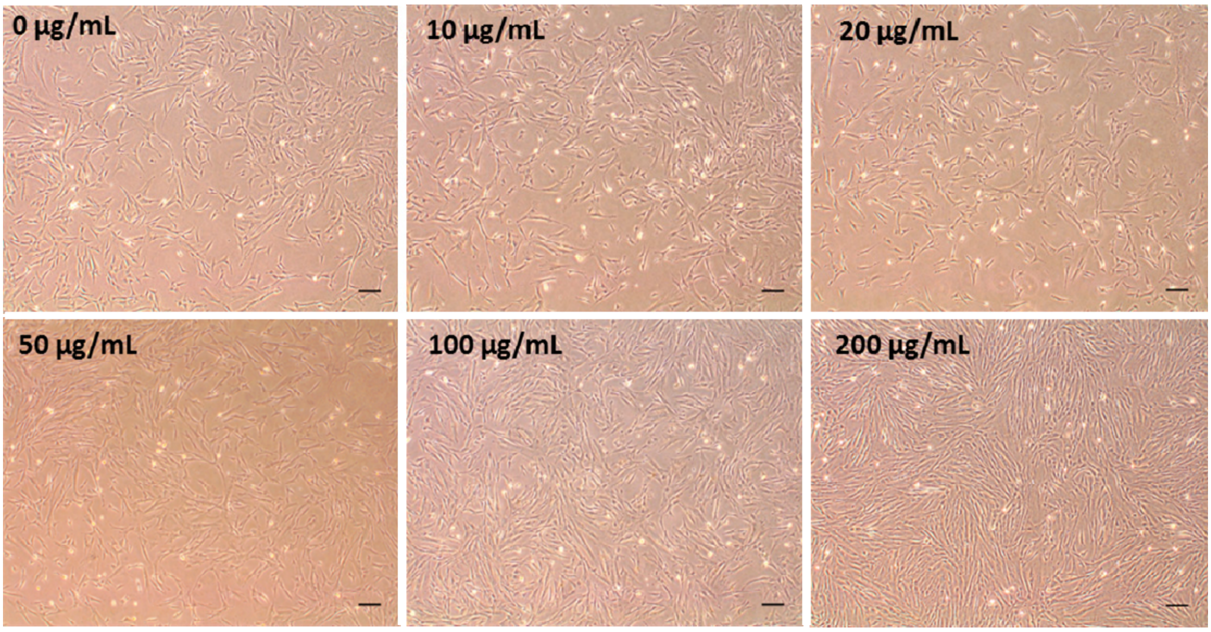

Figure S1: Impact of nanosheet treatment on cellular behavior (Scale bar:50 µm).

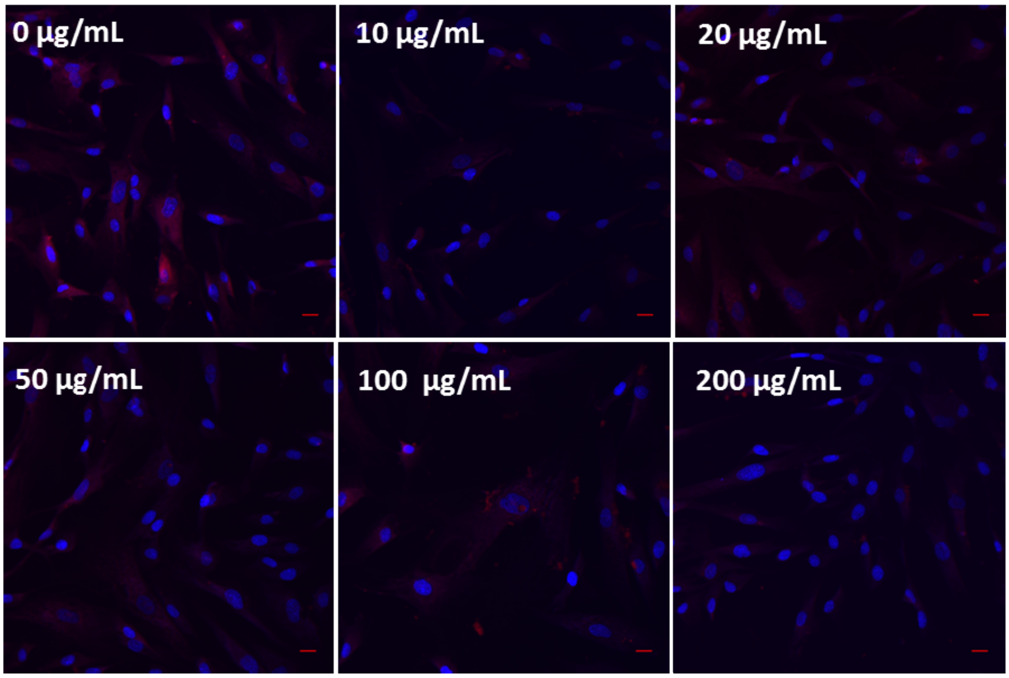

Figure S2: Confocal images of Propidium dye-stained control and treated cells (Scale bar: 50 µm).

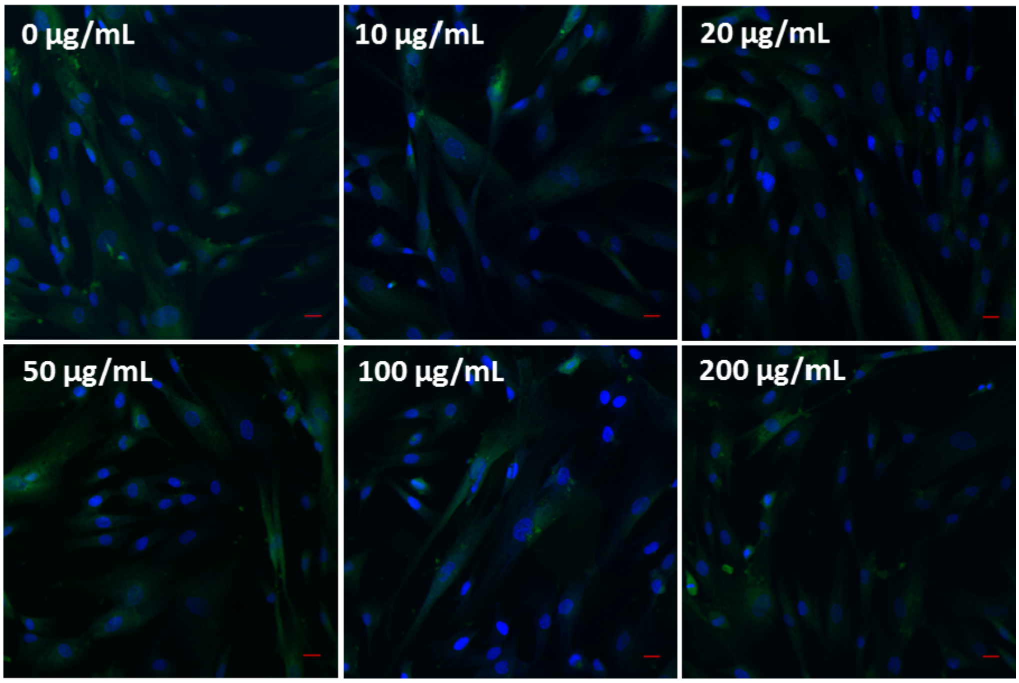

Figure S3: Confocal images of DCF-DA stained control and treated cells (Scale bar:50 μm).

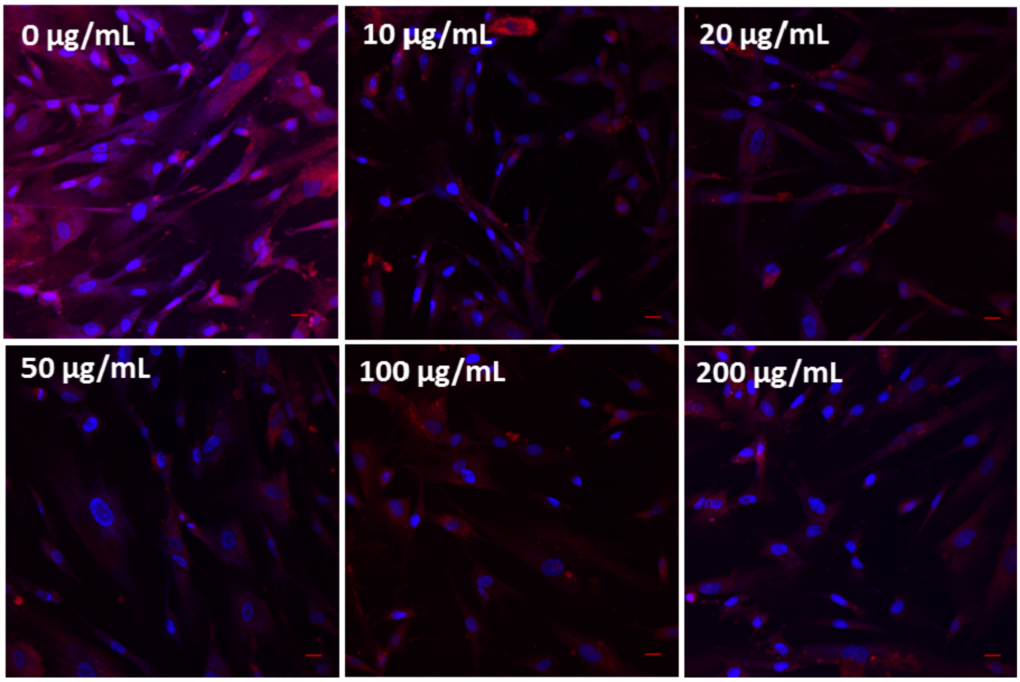

Figure S4: Confocal images of MitoSOX-Red stained control and treated cells (Scale bar: 50 μm).
